# Supplementary material for: p53 dynamics vary between tissues and are linked with radiation sensitivity
Source: Nat Commun. 2021 Feb 9;12:898. doi: 10.1038/s41467-021-21145-z (PMC7873198; doi:10.1038/s41467-021-21145-z)
Supplement: Supplementary file 1 — Supplementary Information [file 41467_2021_21145_MOESM1_ESM.pdf]

Supplementary Information

p53 dynamics vary between tissues and are linked with radiation sensitivity

Jacob Stewart-Ornstein[1,5,], Yoshiko Iwamoto[2], Miles A. Miller[2], Mark A. Prytyskach[2], Stephane Ferretti[3], Philipp Holzer [3], Joerg Kallen[3], Pascal Furet[3], Ashwini Jambhekar[1], William C. Forrester[4], Ralph Weissleder[1,2] and Galit Lahav[1]

[1] Department of Systems Biology and the Ludwig Center at Harvard, Blavatnik Institute at Harvard Medical School, Boston, MA, USA. [2] Center for Systems Biology, Massachusetts General Hospital, Boston, MA, USA. [3] Novartis Institutes for Biomedical Research, Basel, Switzerland. [4] Chemical Biology and Therapeutics, Novartis Institutes for Biomedical Research, Cambridge, MA, USA. [5] Current Address: Department of Computational and Systems Biology, University of Pittsburgh Medical School, Pittsburgh, PA, USA.

Correspondence should be addressed to:
G.L (galit@hms.harvard.edu)
R.W (ralph_weissleder@hms.harvard.edu)

This PDF file includes:

Supplementary Figures 1–7

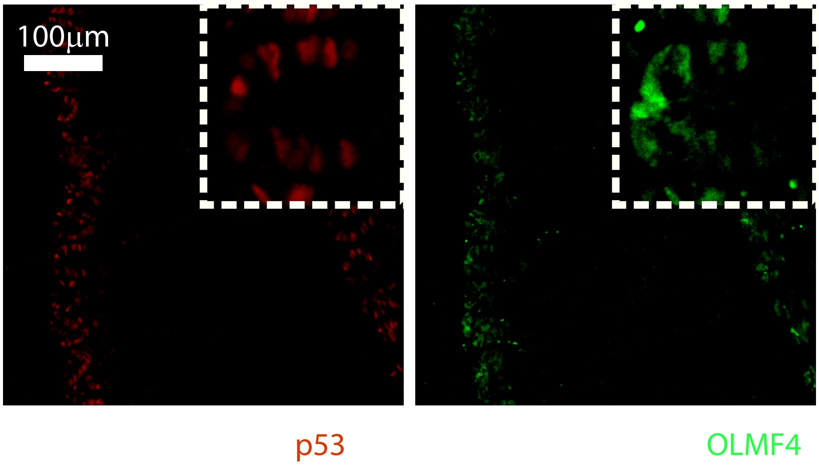

Supplementary Figure 1. Staining of intestines for p53 and the stem cell marker OLMF4. Representative images of intestinal crypts stained for OLMF4 and p53 2hrs after irradiation. Staining was performed on tissues from three mice.

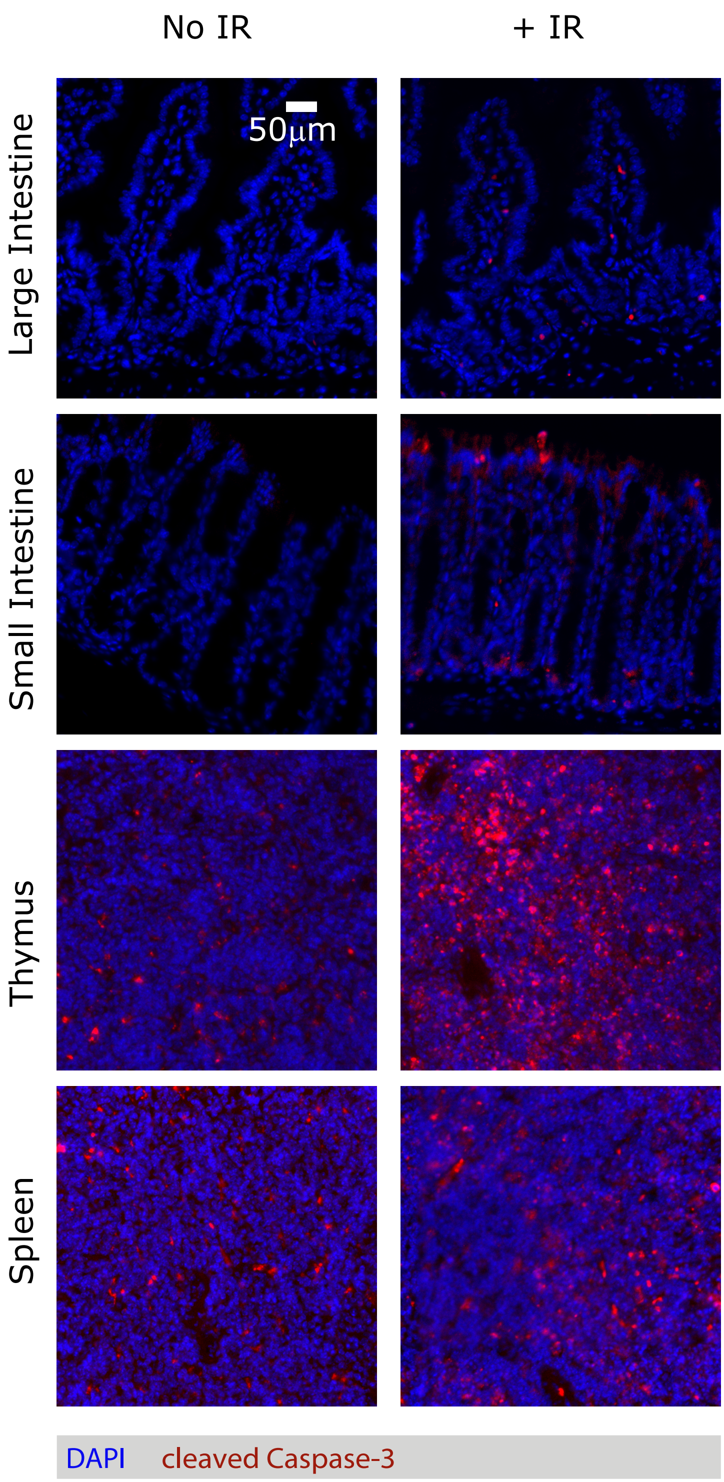

Supplementary Figure 2. Activated Caspase-3 in mouse tissues after radiation. Representative images of the indicated tissues stained for cleaved Caspase-3 prior to irradiation (No IR) and 5hrs after irradiation (+IR). Staining was performed on tissues from three mice.

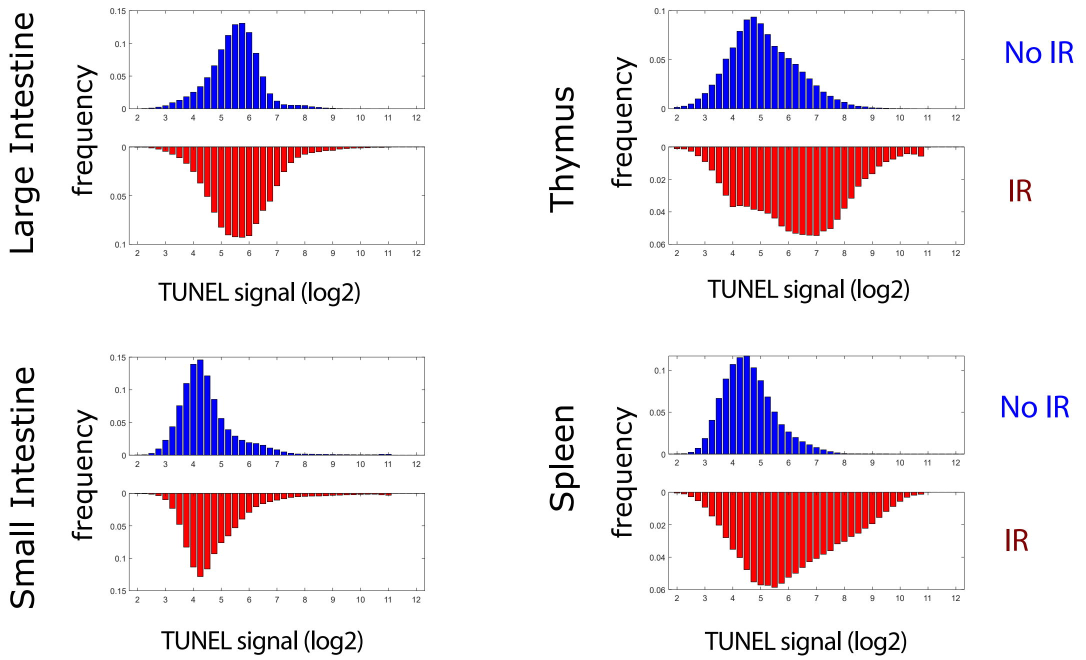

Supplementary Figure 3. Quantification of apoptotic cells by TUNEL staining in mouse tissues after radiation. Quantification of TUNEL staining from Figure 1h. The distributions of TUNEL intensities in untreated (No IR, blue) and irradiated (IR, red) tissues are shown.

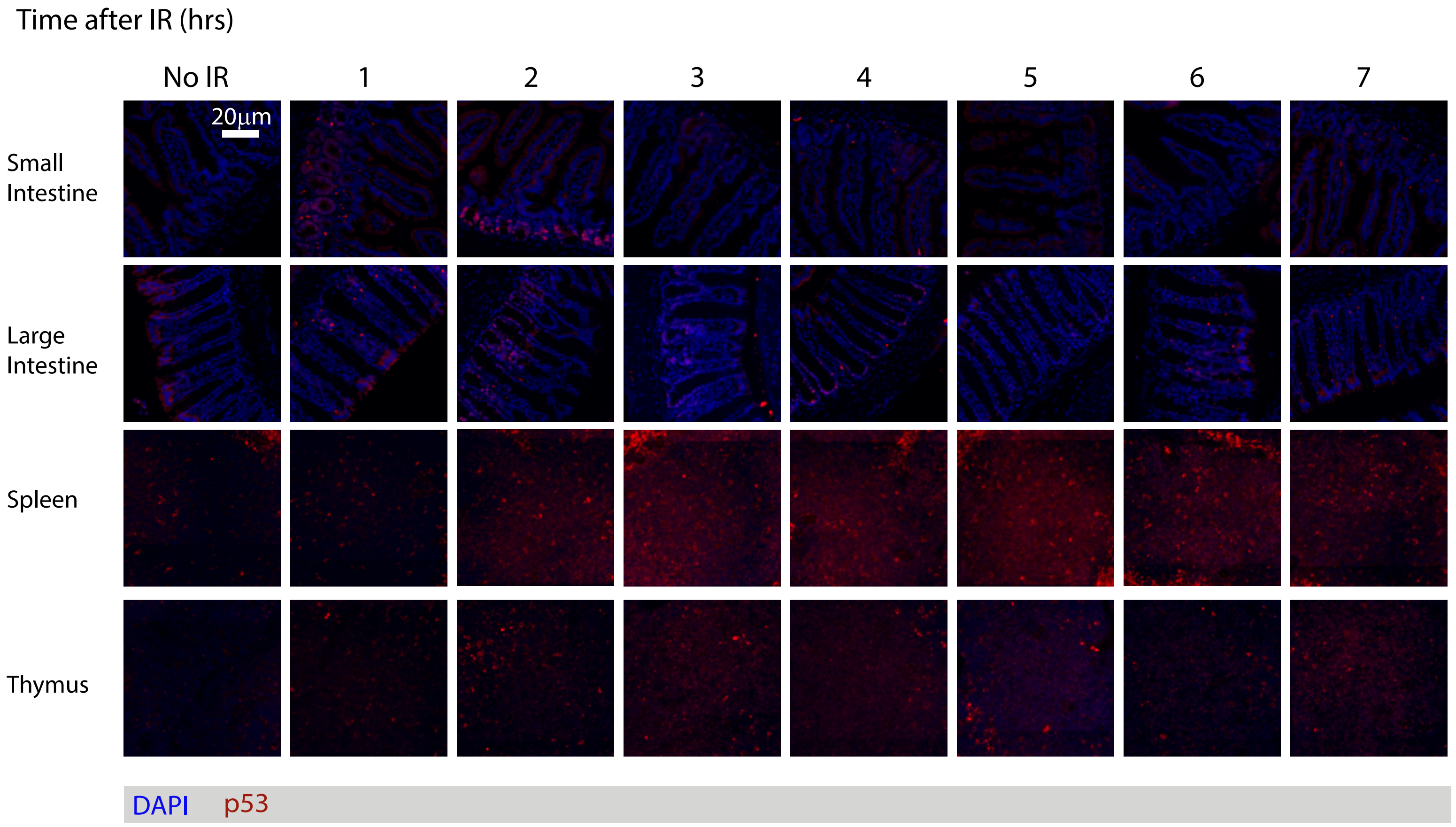

Supplementary Figure 4. p53 intensities in tissues post radiation. Mice were treated with radiation (IR) and p53 levels were measured over time in the indicated tissues by immunofluorescence. Experiment was preformed once as a time-course.

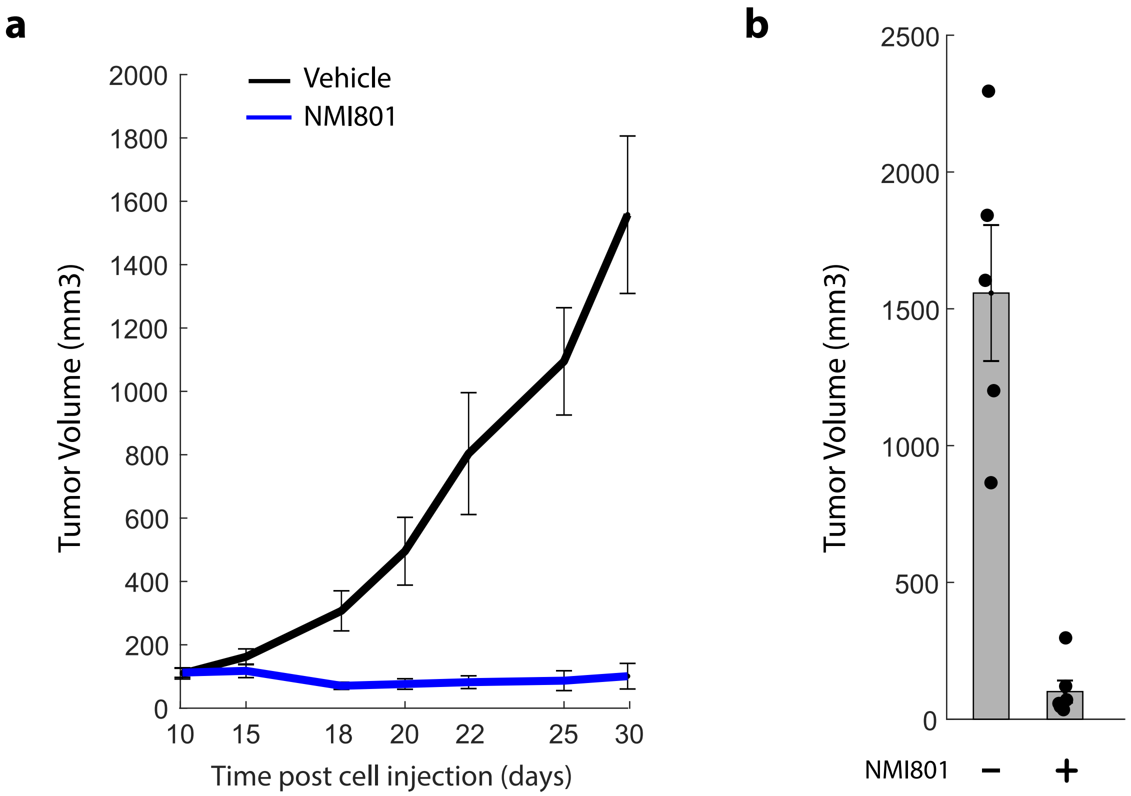

Supplementary Figure 5. Effects of NMI801 on growth of xenograft SJSA-1 tumors. Xenograft tumors of SJSA-1 were allowed to engraft for 13 days to a size of ~100 mm^3. Mice were then treated with NMI801 daily (150 mg/kg) (**a**) Quantification of tumor size at the indicated time points after treatment with vehicle (black line) or NMI801 (blue line) (n= 6 mice per condition; Error bar are SEM). (**b**) Final tumor sizes from data in (a); Dots indicate individual data points; Error bars are SEM; n=5 mice for vehicle and n=6 for NMI801 treated mice.

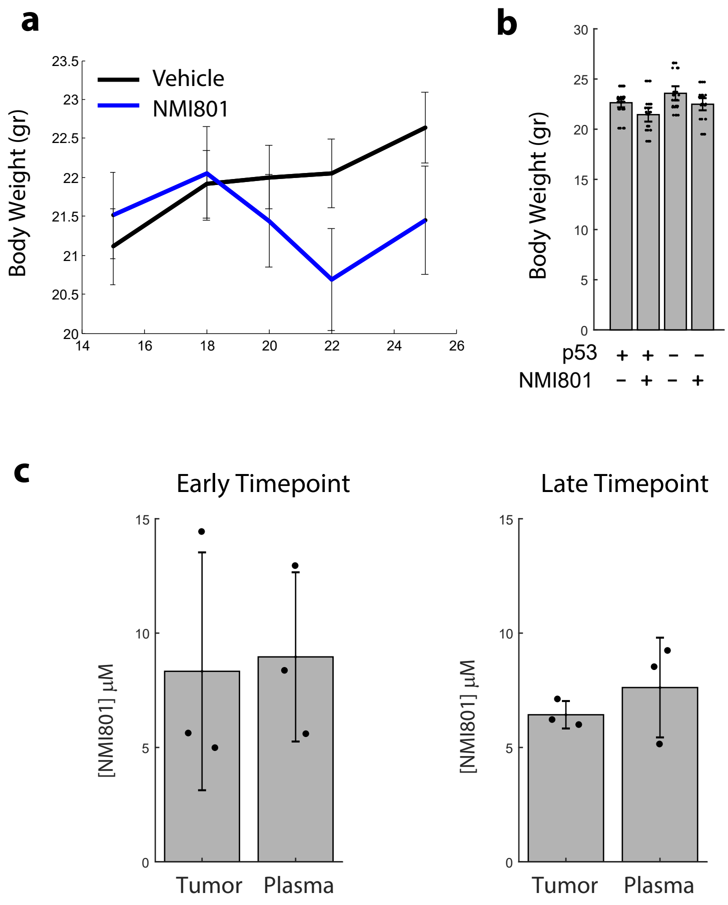

Supplementary Figure 6. Tolerance and distribution of NMI801 in vivo. (**a**) Body weights of mice bearing xenograft HCT116 tumors were measured after treatment with vehicle (black line) or NMI801 (blue line) as described in Fig. 3F. (**b**) Body weights of p53 wild-type (+) or null (-) mice treated with vehicle or NMI801 at the end of the 10-day treatment. (**c**) Pharmacokinetic measurements of NMI801 concentration in the tumor and plasma 3 hrs after the first treatment (early time point) or 3 hrs after the last treatment (late time point). Y axis values are normalized between tumor and plasma compartments by conversion factor; 1μM (plasma) = 1nMol/gr for tumor weight. (n= 7 mice for a, b; n= 3 mice for c; Error bars are SEM; Dots indicate individual data points).

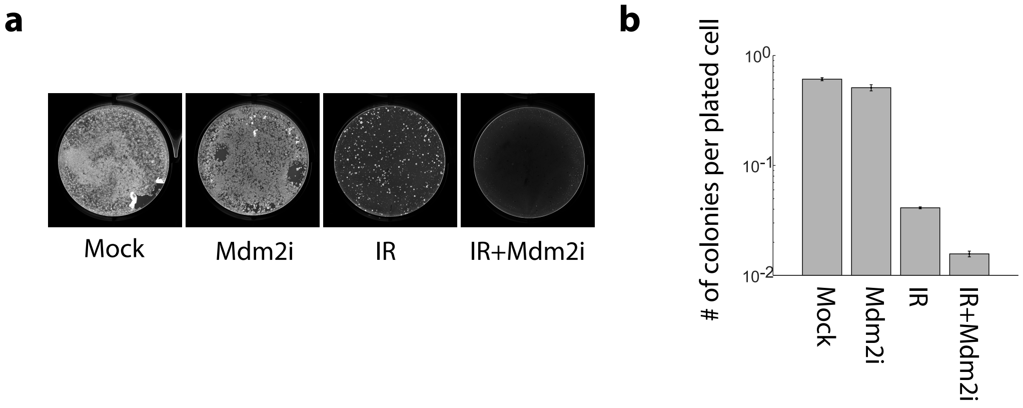

Supplementary Figure 7. Effects of radiation and NMI801 on colony formation of cultured HCT116 colon cancer cells. HCT116 cells (2.5 x 10^5) were plated and either left untreated or were treated with irradiation alone (IR, 5Gy), NMI801 (Mdm2i, 1μM) alone, or a combination of radiation and Mdm2i. (**a**) Two days after treatment cells were split and diluted 1:64. Plates were fixed and imaged after 7 days. (**b**) Quantification of colony numbers from (a) using serial dilutions. Experiments were done in triplicates. Error bars are SEM.
