# Supplementary material for: p53 dynamics vary between tissues and are linked with radiation sensitivity
Source: Nat Commun. 2021 Feb 9;12:898. doi: 10.1038/s41467-021-21145-z (PMC7873198; doi:10.1038/s41467-021-21145-z)
Supplement: Supplementary file 3 — Reporting Summary [file 41467_2021_21145_MOESM3_ESM.pdf]

nature research

Corresponding author(s): Galit Lahav

Last updated by author(s): Dec 11, 2020

Reporting Summary

Nature Research wishes to improve the reproducibility of the work that we publish. This form provides structure for consistency and transparency in reporting. For further information on Nature Research policies, see our Editorial Policies and the Editorial Policy Checklist.

Statistics

For all statistical analyses, confirm that the following items are present in the figure legend, table legend, main text, or Methods section.

n/a	Confirmed	
☐	☒	The exact sample size (*n*) for each experimental group/condition, given as a discrete number and unit of measurement
☒	☐	A statement on whether measurements were taken from distinct samples or whether the same sample was measured repeatedly
☐	☒	The statistical test(s) used AND whether they are one- or two-sided<br>*Only common tests should be described solely by name; describe more complex techniques in the Methods section.*
☒	☐	A description of all covariates tested
☒	☐	A description of any assumptions or corrections, such as tests of normality and adjustment for multiple comparisons
☐	☒	A full description of the statistical parameters including central tendency (e.g. means) or other basic estimates (e.g. regression coefficient) AND variation (e.g. standard deviation) or associated estimates of uncertainty (e.g. confidence intervals)
☐	☒	For null hypothesis testing, the test statistic (e.g. *F*, *t*, *r*) with confidence intervals, effect sizes, degrees of freedom and *P* value noted<br>*Give P values as exact values whenever suitable.*
☒	☐	For Bayesian analysis, information on the choice of priors and Markov chain Monte Carlo settings
☒	☐	For hierarchical and complex designs, identification of the appropriate level for tests and full reporting of outcomes
☒	☐	Estimates of effect sizes (e.g. Cohen's *d*, Pearson's *r*), indicating how they were calculated

Our web collection on statistics for biologists contains articles on many of the points above.

Software and code

Policy information about availability of computer code

Data collection	NIS elements was used to collect images for histology.
Data analysis	Analysis was preformed using ImageJ (image anlysis and presentation), Excel (qPCR data handling and statistical tests), and matlab (for statistical tests and image analysis). Analyses are described in the methods. Further details on image analysis are available on request.

For manuscripts utilizing custom algorithms or software that are central to the research but not yet described in published literature, software must be made available to editors and reviewers. We strongly encourage code deposition in a community repository (e.g. GitHub). See the Nature Research guidelines for submitting code & software for further information.

Data

Policy information about availability of data

All manuscripts must include a data availability statement. This statement should provide the following information, where applicable:

- Accession codes, unique identifiers, or web links for publicly available datasets
- A list of figures that have associated raw data
- A description of any restrictions on data availability

Included

Field-specific reporting

Please select the one below that is the best fit for your research. If you are not sure, read the appropriate sections before making your selection.

☒ Life sciences ☐ Behavioural & social sciences ☐ Ecological, evolutionary & environmental sciences

For a reference copy of the document with all sections, see nature.com/documents/nr-reporting-summary-flat.pdf

Life sciences study design

All studies must disclose on these points even when the disclosure is negative.

Sample size	No power calculation were done. Sample sizes were selected based on prior experience as to sufficient animals/data for robust statistical results.
Data exclusions	No data was excluded.
Replication	Experimental results were reproducible. Replicate numbers are indicated in the figure legends.
Randomization	Mice were randomized for treatment by cage.
Blinding	Researchers were not blinded at any stage.

Reporting for specific materials, systems and methods

We require information from authors about some types of materials, experimental systems and methods used in many studies. Here, indicate whether each material, system or method listed is relevant to your study. If you are not sure if a list item applies to your research, read the appropriate section before selecting a response.

Materials & experimental systems

n/a	Involved in the study	
☐	☒	Antibodies
☐	☒	Eukaryotic cell lines
☒	☐	Palaeontology and archaeology
☐	☒	Animals and other organisms
☒	☐	Human research participants
☒	☐	Clinical data
☒	☐	Dual use research of concern

Methods

n/a	Involved in the study	
☒	☐	ChIP-seq
☒	☐	Flow cytometry
☒	☐	MRI-based neuroimaging

Antibodies

Antibodies used	p53: CM5 (Leica) for mouse tissue, p53: FL393 (Santa Cruz, discontinued) p53: 7F5 (Cell Signaling) for tumors. Gamma H2A.X (phospho S139) was stained with Abcam antibody EP854(2)Y and H2AX phospho – AF488 (2F3, Biolegend) for co-staining with p53. Olfm4 (D6Y5A) was directly conjugated to AF488 (Cell Signaling) for co-staining with p53. mki67-FITC (SOLa15, Thermofisher). Apoptosis was detected with anti-Cleaved Caspase-3 (cell signaling #9661). The p53 target gene p21 (Abcam, EPR362). Phospho-p53 (S20) was stained with a polyclonal antibody from Invitrogen (PA5-104741)
Validation	p53 antibodies were validated by western by the suppliers. EP854(2)Y and 2F7 were validated by western and immunoflorescence using DNA damage treated positive control samples. OLMF4 (D6Y5A) was validated by western and IHC by cell signaling. Ki67 (SOLa15) was validated by histology and western blot by the supplier. Anti-Cleaved Caspase-3 (cell signaling #9661) was validated by western and histology of DNA damage treated samples by the supplier. Anti-p21 Abcam, EPR362 was validated by western blot, histology, and immunoflorescence by the supplier. Anti-p-p53 (PA5-104741) was validated by westernblot, IHC, and immunoflorescence by the supplier.

Eukaryotic cell lines

Policy information about cell lines

Cell line source(s)	HCT116 (ATCC), HEPA-1C1cC7 (a gift from Weitz Lab, Harvard Medical School), HCT116-deltap53 (a gift from Vogelstein lab, John Hopkins), SJSA-1 (ATTC).
Authentication	Str-Profiling of HCT116 cells
Mycoplasma contamination	HCT116 cells were tested for mycoplasma contamination before animal experiments

Commonly misidentified lines (See ICLAC register)	none

Animals and other organisms

Policy information about studies involving animals; ARRIVE guidelines recommended for reporting animal research

Laboratory animals	C57BL/6, Nu/Nu mice
Wild animals	none
Field-collected samples	none
Ethics oversight	(1) Animal studies on NMI801 were conducted in accordance with ethics and procedures covered by permit nos. BS-1975 issued by the Kantonales Veterinäramt Basel-Stadt and in strict adherence to guidelines of the Eidgenössisches Tierschutzgesetz and the Eidgenössische Tierschutzverordnung, Switzerland<br>(2) Animal research involving irradiation was performed in accordance with guidelines from the Institutional Subcommittee on Research Animal Care and with approval of the Institutional Animal Care and Use Committee at the Massachusetts General Hospital Research Institute.

Note that full information on the approval of the study protocol must also be provided in the manuscript.
